# Symptom Improvement in Chronic Subjective Tinnitus Following Transcranial Magnetic Stimulation: A Retrospective Open-Label Study

**DOI:** 10.1192/j.eurpsy.2026.10711

**Published:** 2026-07-31

**Authors:** A. S. Verma, R. Gupta, B. Bansal, A. Pathak, N. Kumar, V. Patil

**Affiliations:** 1 Psychiatry, Case Western Reserve University/ Metrohealth, Cleveland; 2 Psychiatry, Brigham & Women’s Faulkner Hospital; 3 Psychiatry, Harvard Medical School, Boston, United States; 4 AIIMS, New Delhi, India; 5 Psychiatry, Cleveland Clinic Main Campus, Cleveland, United States; 6 Psychiatry, AIIMS, New Delhi, India

## Abstract

**Introduction:**

Tinnitus, particularly in cases without an identifiable physical cause, often poses a significant treatment challenge, with limited effectiveness of pharmacological interventions.Transcranial Magnetic Stimulation (TMS), has shown promise in alleviating tinnitus symptoms

**Objectives:**

To evaluate the effectiveness of TMS in reducing severity of Chronic subjective Tinnitus, as measured by changes in Tinnitus Handicap Inventory (THI) scores

**Methods:**

This retrospective study involved 32 adult patients diagnosed with tinnitus, who received TMS. The number of treatments (10) and the follow-up duration (4 weeks) were chosen based on standardized treatment guidelines and tailored to patients’ needs.-The assessment included pre-and post-treatment evaluations using the THI (Tinnitus Handicap Inventory) to measure changes in patient-reported tinnitus severity. The Wilcoxon rank sum test was used to compare the THI scores among patients at baseline and 4 weeks of follow-up

**Results:**

Our participants were predominantly men (72%) with a median age of 36 years (IQR: 28-51 years) and a median disease duration of 2.0 years (IQR:0.7-3.3 years). Significant improvement was observed in the THI scores, decreasing from a median of 53 (IQR: 34-67) at baseline to 35 (IQR: 24-57) post-treatment (p-value = 0.0151). Most treatments were conducted at 1 Hz frequency (79%). Around one-fifth of the patients were on benzodiazepines

**Conclusions:**

This study provides evidence for the effectiveness of TMS for tinnitus. We are hopeful that future studies would replicate our findings and efforts would be directed at optimizing TMS protocols for its more widespread use in treating tinnitus.

**Disclosure of Interest:**

None Declared

